# The Periaqueductal Gray (PAG)

**DOI:** 10.1155/2009/360907

**Published:** 2009-04-01

**Authors:** T. A. Lovick, Robert Adamec

**Affiliations:** ^1^College of Medical and Dental Sciences, University of Birmingham, Birmingham B15 2TT, UK; ^2^Department of Psychology, Memorial University of Newfoundland, St. John's, Newfoundland, Canada A1B 3X9

This issue covers a broad territory of PAG function: neuropharmacology, functional organization, and PAG plasticity in adaptive behavior, emotion, anxiety, and the less-studied plasticity of the PAG function in females.

Interest in the involvement of PAG in defensive behavior has a long history. Thinking about this area was radically changed by the seminal contributions of Bandler and DePaulis in defining functional columns arranged in both coronal and saggital planes of the PAG. Most papers in this section respect the importance of those functional columns while adding to our understanding how they might contribute to panic, anxiety, defensive response to natural threats, as well as aversive learning.

Del-Ben and Graeff in the paper entitled “Panic disorder: Is the PAG involved?” review preclinical as well as human imaging research in the contribution of PAG dysfunction to panic anxiety disorder. Others have shown how preclinical models of posttraumatic stress disorder (PTSD) and using brief exposure to predators (predator stress) have helped to advance our understanding of the neural substrates of stress-induced lasting sensitization of rodent anxiety including potentiation of startle response. Adamec et al. contribution in the paper entitled “Viral Vector Induction of CREB Expression in the Periaqueductal Gray Induces a Predator Stress-Like Pattern of Changes in pCREB Expression, Neuroplasticity and Anxiety in Rodents” extends initial findings implicating pCREB expression in the lateral column of the PAG in neuroplastic changes in amygdala-PAG communication as mediators of predator stress-enhanced startle and anxiety. Using viral vector enhancement of CREB expression in the PAG, the authors provide support for the idea that stress-precipitated increases in pCREB in the PAG are sufficient to potentiate central amygdala to PAG neural transmission, to increase anxiety in the elevated plus maze, and to potentiate startle response. In a related vein, exposure to stressful stimuli increases endocannabinoid (eCB) levels in the PAG and local administration of cannabinoid receptor 1 (CB1) agonists or drugs that facilitate eCB-mediated neurotransmission produce antinociceptive and antiaversive effects. Stimulated by these findings, Moreira et al. in “Anti-aversive effects of cannabinoids: is the periaqueductal gray involved?” explore the anxiolytic potential of PAG injection of CB1 agonists and cannabidiol in a variety of tests of anxiety as well as in contextual fear conditioning. They provide evidence that dorsal lateral PAG is a site of antiaversive actions of cannabinoids. Other PAG columnar function (ventrolateral PAG-VlPAG) in antinociception is explored by Morgan et al. in “Behavioral Consequences of Delta Opioid Receptor Activation in the Periaqueductal Gray of Morphine Tolerant Rats”. It is known that chronic morphine administration shifts delta-opioid receptors (DOR) from the cytoplasm to the plasma membrane. Moreover, microinjection of morphine into the VlPAG produces antinociception. In light of these findings, it was hypothesized that movement of DORs to the membrane would induce antinociception to the DOR agonist deltorphin II as a way to compensate for morphine tolerance. Their findings suggest that chronic morphine administration alters DORs in the vlPAG with little evidence of compensation for the decrease in antinociception caused by morphine tolerance.

*PAG Function in Females*. Until very recently, research on the PAG along with most other brain areas was conducted almost exclusively in males with the implicit assumption that the anatomy and physiology were the same in females. Three articles in this issue show that this is not the case. Not only are there significant differences in the organization of the PAG between the sexes but the circuitry of the female PAG also exhibits considerable plasticity in response to changes in its hormonal milieu.

Lloyd and Murphy in the paper entitled “The Role of the Periaqueductal Gray in the Modulation of Pain in Males and Females: Is the Anatomy and Physiology Really That Different?” show that descending projections from the PAG, which are believed to modulate spinal processing of nociceptive input, are more numerous in female rats than in males. Paradoxically morphine, which exerts much of its antinociceptive effects by activating these projections, excited a much smaller proportion of the neurones in females compared to males, a finding that may explain the clinical finding of reduced efficacy of the analgesic effects of morphine in women compared to men. Lovick and Devall in the paper entitled “The Role of the Periaqueductal Gray in the Modulation of Pain in Males and Females: Is the Anatomy and Physiology Really That Different?” show how the fall in production of progesterone during the late dioestrus phase of the oestrous cycle leads to upregulation of certain subunits of the GABA_A_ receptor in the PAG. One of the functional consequences is a decrease in ongoing GABAergic tone and an increase in neural excitability within PAG circuits. These changes may underlie the increased susceptibility to the development of stress-induced hyperalgesia that was shown to occur during late dioestrus and may also be relevant to Lloyd and Murphy's reports of a reduction in morphine's efficacy in female rats during dioestrus. Mota-Ortiz et al. in the paper entitled “Afferent Connections to the Rostrolateral Part of the Periaqueductal Gray: a Critical Region Influencing the Motivation Drive to Hunt and Forage” examine afferent inputs to the rostrolateral PAG (rlPAG ) in female rats. In nursing females, morphine treatment induces a behavioral “switch” from maternal to foraging behavior which is mediated via the rostrolateral PAG (rlPAG ). The authors report that the rlPAG receives inputs from medial prefrontal cortical areas involved in controlling attention-related and decision-making processes. Other afferents from different amygdalar, hypothalamic, and brainstem sites provide information to PAG related to feeding, drinking, or hunting behaviors. It is suggested that this unique combination of afferent connections positions the rlPAG to influence the decision whether hunting/foraging or other behaviors would be the most appropriate adaptive response for females, particularly in the presence of their young.

These papers are by no means exhaustive of the interest and breadth of work in the PAG field. Nevertheless, together they reflect the rich functional diversity of activities of the PAG. Moreover, they make apparent the importance of paying attention to sex and particular columnar areas when studying this fascinating structure, whose activity is of fundamental importance for survival of the individual in a challenging and constantly changing environment.

*T. A. Lovick* *T. A. Lovick* *Robert Adamec* *Robert Adamec*

